# Multipressure Sampling for Improving the Performance of MOF-based Electronic Noses

**DOI:** 10.1021/acssensors.4c00199

**Published:** 2024-07-12

**Authors:** Brian A. Day, Nicolas I. Ahualli, Christopher E. Wilmer

**Affiliations:** † Department of Chemical and Petroleum Engineering, University of Pittsburgh, Pittsburgh, Pennsylvania 15261, United States; ‡ Department of Electrical and Computer Engineering, University of Pittsburgh, Pittsburgh, Pennsylvania 15261, United States; § Clinical and Translational Science Institute, University of Pittsburgh, Pittsburgh, Pennsylvania 15213, United States

**Keywords:** metal−organic framework, chemical sensing, electronic nose, molecular simulations, methane, benzene, hydrogen sulfide

## Abstract

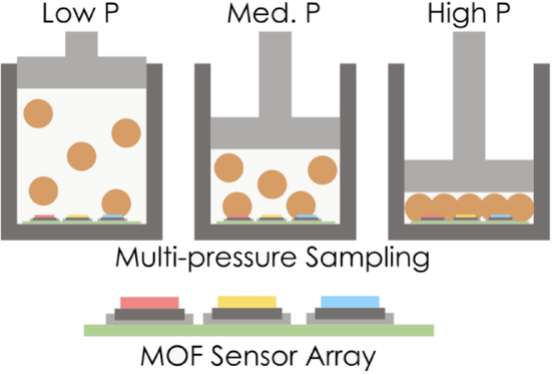

Metal–organic frameworks (MOFs) are a promising class of porous materials for the design of gas sensing arrays, which are often called electronic noses. Due to their chemical and structural tunability, MOFs are a highly diverse class of materials that align well with the similarly diverse class of volatile organic compounds (VOCs) of interest in many gas detection applications. In principle, by choosing the right combination of cross-sensitive MOFs, layered on appropriate signal transducers, one can design an array that yields detailed information about the composition of a complex gas mixture. However, despite the vast number of MOFs from which one can choose, gas sensing arrays that rely too heavily on distinct chemistries can be impractical from the cost and complexity perspective. On the other hand, it is difficult for small arrays to have the desired selectivity and sensitivity for challenging sensing applications, such as detecting weakly adsorbing gases with weak signals, or conversely, strongly adsorbing gases that readily saturate MOF pores. In this work, we employed gas adsorption simulations to explore the use of a variable pressure sensing array as a means of improving both sensitivity and selectivity as well as increasing the information content provided by each array. We studied nine different MOFs (HKUST-1, IRMOF-1, MgMOF-74, MOF-177, MOF-801, NU-100, NU-125, UiO-66, and ZIF-8) and four different gas mixtures, each containing nitrogen, oxygen, carbon dioxide, and exactly one of the hydrogen, methane, hydrogen sulfide, or benzene. We found that by lowering the pressure, we can limit the saturation of MOFs, and by raising the pressure, we can concentrate weakly adsorbing gases, in both cases, improving gas detection with the resulting arrays. In many cases, changing the system pressure yielded a better improvement in performance (as measured by the Kullback–Liebler divergence of gas composition probability distributions) than including additional MOFs. We thus demonstrated and quantified how sensing at multiple pressures can increase information content and cross-sensitivity in MOF-based arrays while limiting the number of unique materials needed in the device.

Over the past few years, there has been renewed interest in gas sensing arrays, also known as electronic noses, due to improvements in both sensing materials and advances in computational analysis techniques.^1−6^ In particular, research in the field of electronic noses has benefited from the explosive growth of research in metal–organic frameworks (MOFs) over the past two decades. MOFs are a large class of chemically and structurally diverse nanoporous crystalline materials with very high internal surface areas.^7,8^ They exhibit impressive and diverse gas adsorption properties, which make them promising sensing elements in electronic noses, where high signal-to-noise ratios and cross-sensitivity are needed.^9^ To date, over 9000 different MOFs have been synthesized, and over 500 000 MOFs have been predicted.^10,11^ Due to the availability of large databases for MOF materials, research in MOF-based gas sensing has become less focused on material discovery and more focused on material selection, with a common approach being to functionalize carefully selected MOFs to fine-tune adsorption behaviors and improve sensing.^12−15^ These approaches are accelerated using computational techniques for modeling gas adsorption, such as grand-canonical Monte Carlo (GCMC) simulations, which makes the initial selection of materials much easier.^11^

Compared with gas chromatography–mass spectrometry (GCMS), electronic noses tend to be low-cost, highly portable devices with fast response times, but at the expense of sensitivity and selectivity.^16−18^ Even so, these features open many application areas, including industrial process monitoring, environmental monitoring, security, and of particularly high interest, disease diagnostics.^5,19−21^ The challenge for electronic noses is improving the performance without sacrificing other desirable features such as speed and portability.

The performance of sensor arrays can be improved, in principle, by the addition of more complementary sensing elements.^4,22,23^ For this reason, much of our prior work has been focused on selecting the best materials for many-element sensor arrays and examining how the performance of the devices improves with the array size.^24−27^ These electronic noses can be composed of MOF films deposited onto mass-responsive sensors, such as quartz crystal microbalances (QCM) or surface acoustic wave (SAW) devices, with the change in mass due to adsorption being measurable with nanogram and greater sensitivity.^16,28^ With the knowledge of how the adsorbed mass changes as a function of the bulk gas composition for each MOF in an array, we can determine the composition of an unknown gas mixture from only the detected mass values, that is, assuming that the array has the needed signal-to-noise ratios and cross-sensitivities. In prior works, we examined a set of 50 MOFs for methane-in-air sensing, carbon dioxide-in-air sensing, and also ammonia-in-breath sensing for kidney disease detection.^26,27,29,30^ However, for more complex gas mixtures (i.e., more components and lower concentrations of important gases), it can become increasingly difficult to find MOFs with the adsorption behaviors needed to design a high-performing array.

In this work, we explored the impact that multipressure sampling can have on the performance of gas sensing arrays. We examined a set of nine MOFs (HKUST-1, IRMOF-1, MgMOF-74, MOF-177, MOF-801, NU-100, NU-125, UiO-66, and ZIF-8) and four gases (benzene, methane, hydrogen, and hydrogen sulfide) in a mixture of N_2_/O_2_/CO_2_ across five system pressures (0.1, 0.5, 1.0, 5.0, and 10.0 bar). By designing arrays in which we employ both multiple MOFs and pressures, we can detect previously challenging gas compositions and increase the information content of the device without the need to increase the number of MOFs. To our knowledge, no one has yet discussed and quantified the benefit of building a sensor array that samples a gas mixture at *multiple* pressures as a way of improving sensing.

## Methods

In this work, we examined a set of nine MOFs (HKUST-1, IRMOF-1, MgMOF-74, MOF-177, MOF-801, NU-100, NU-125, UiO-66, and ZIF-8), which we had used in previous studies on the design of electronic noses, chosen for being well-studied, synthesizable MOFs exhibiting chemical and structural diversities. The molecular modeling software RASPA was used to run GCMC simulations of gas adsorption in these MOFs at a temperature of 298 K and various pressures (0.1, 0.5, 1.0, 5.0, and 10 bar).^31^ We simulated four different sets of four-component gas mixtures: benzene in N_2_/O_2_/CO_2_, methane in N_2_/O_2_/CO_2_, hydrogen in N_2_/O_2_/CO_2_, and hydrogen sulfide in N_2_/O_2_/CO_2_. These four gases, along with CO_2_, were varied from 0 to 1% in 0.05% increments. No mixtures contained more than one of benzene, methane, hydrogen, or hydrogen sulfide. N_2_/O_2_ made up the remaining gas mixture (to maintain atmospheric pressure), always in a 4:1 ratio (to represent ambient air), for a total of 441 unique compositions per gas mixture. A detailed description of the simulations, including full parameter sets and raw simulation data, is provided in the Supporting Information. Note that in some figures, the simulated adsorbed mass data have noticeable noise; this is due to the low number of Monte Carlo steps used in the simulations to accommodate the large number of compositions needed to simulate. This effect is particularly pronounced at low pressures with limited gas adsorption for which the signal-to-noise ratio is quite poor and underscores the benefit of sampling at other pressures. Additionally, note that the scale for the color bars changes for different gases and pressures, as the range of data were too large to effectively see trends while employing a single color bar for all plots.

After generating a complete set of adsorption data for all gas mixtures, MOFs, and pressures, we designed and evaluated various arrays. We created two different types of arrays in this work: single-pressure arrays, in which we sample the gas mixture at only one of the simulated pressures, and all-pressure arrays, in which we sample the gas mixture at all simulated pressures. With nine MOFs, there are only 511 possible unique arrays; so, both single- and all-pressure cases could be explored comprehensively (i.e., via brute force).

A brief overview of the method used for predicting gas compositions and quantifying array performance is given below, and a more detailed description can be found in the Supporting Information and our previous works.^24−27,29^ To begin the analysis, we must first generate a set of detected masses for each of the arrays, here simply using the simulated adsorption data for each sensing element at a known composition, in this case, 79% N_2_, 19.75% O_2_, 0.5% CO_2_, and 0.75% of the other gases of interest. Since our goal is to predict the composition given the set of detected masses, we “forget” the composition which was used to generate the detected masses. Next, using a truncated normal distribution with a standard deviation of 1 mg/g-framework for hydrogen, methane, and hydrogen sulfide, and 10 mg/g-framework for benzene (because of higher simulation error), we generate a set of probabilities for all compositions based on how close the detected and predicted masses are to each other. In this regard, we have high confidence in the detected mass when the adsorbed mass is much greater than the device noise, and our prediction when the change in the adsorbed mass as a function of composition is sharp. Conversely, even if we have high confidence in the detected mass, we cannot accurately predict the gas composition if the adsorbed mass does not change as a function of composition.

Lastly, the array performance was quantified using a metric known as the Kullback–Liebler divergence (KLD), which effectively scores the quality of the predicted composition when compared to the random chance, with a higher score corresponding to a more certain prediction.^32,33^ The maximum possible KLD is limited by the number of possible compositions. For 441 unique compositions, the maximum possible value is 6.089 bits. The equation for KLD can be found in the Supporting Information.

## Results

### High Pressure Example (Methane/Hydrogen)

Methane and hydrogen are both small nonpolar molecules and, as a result, are typically weakly adsorbing gases. Consequently, when exposing MOFs to complex gas mixtures, these gases will frequently make up only a small fraction of the total adsorbed mass, an effect that is exaggerated when these gases are present in very low concentrations. In order to reliably detect and quantify gases with mass-based sensing, it generally helps to increase the amount in which they adsorb relative to those of the other gas species. With this in mind, we hypothesized that by increasing the system pressure, we could increase the amount of gas adsorbed, particularly for small molecules, which should pack more efficiently than large molecules, and thus, improve the ability to detect gases like methane and hydrogen in complex gas mixtures. That being said, the size difference between nitrogen (3.64 Å) and oxygen (3.46 Å) versus hydrogen (2.89 Å) and methane (3.80 Å) is not very significant, compared to a molecule like benzene (5.85 Å), so it is not obvious what impact increased pressures would have on the selectivity of adsorbed gases as the steric effects become more important, especially in the presence of strongly adsorbing small gases like CO_2_.^34^ Fortunately, it is still possible that even if the selectivity does not change by increasing pressure, increasing the total adsorbed mass could improve the performance of mass-based sensors simply by increasing the signal-to-noise ratio.

**Figure 1:**
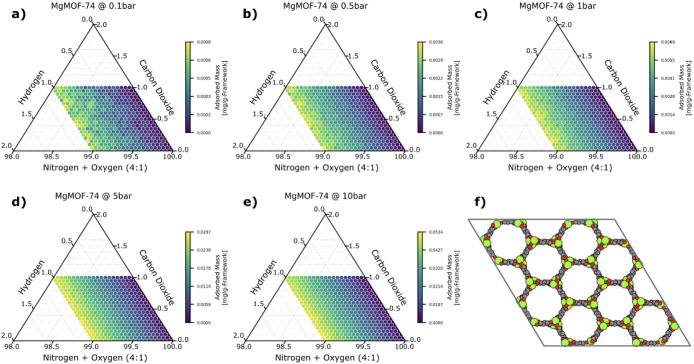
Ternary plots of the adsorbed mass of hydrogen in MgMOF-74 as a function of composition and at the following pressures: a) 0.1 bar, b) 0.5 bar, c) 1 bar, d) 5 bar, and e) 10 bar. In panel f), we show a 2 × 2 × 2 unit cell of the MOF projected down the *c*-axis. Each axis shows the mole fraction of the corresponding gas (a 4:1 ratio for the nitrogen:oxygen axis), extending along the orientation of the tick marks. The color of each point in the ternary plot corresponds to the adsorbed mass of hydrogen in units of mg/g-framework.

**Figure 2:**
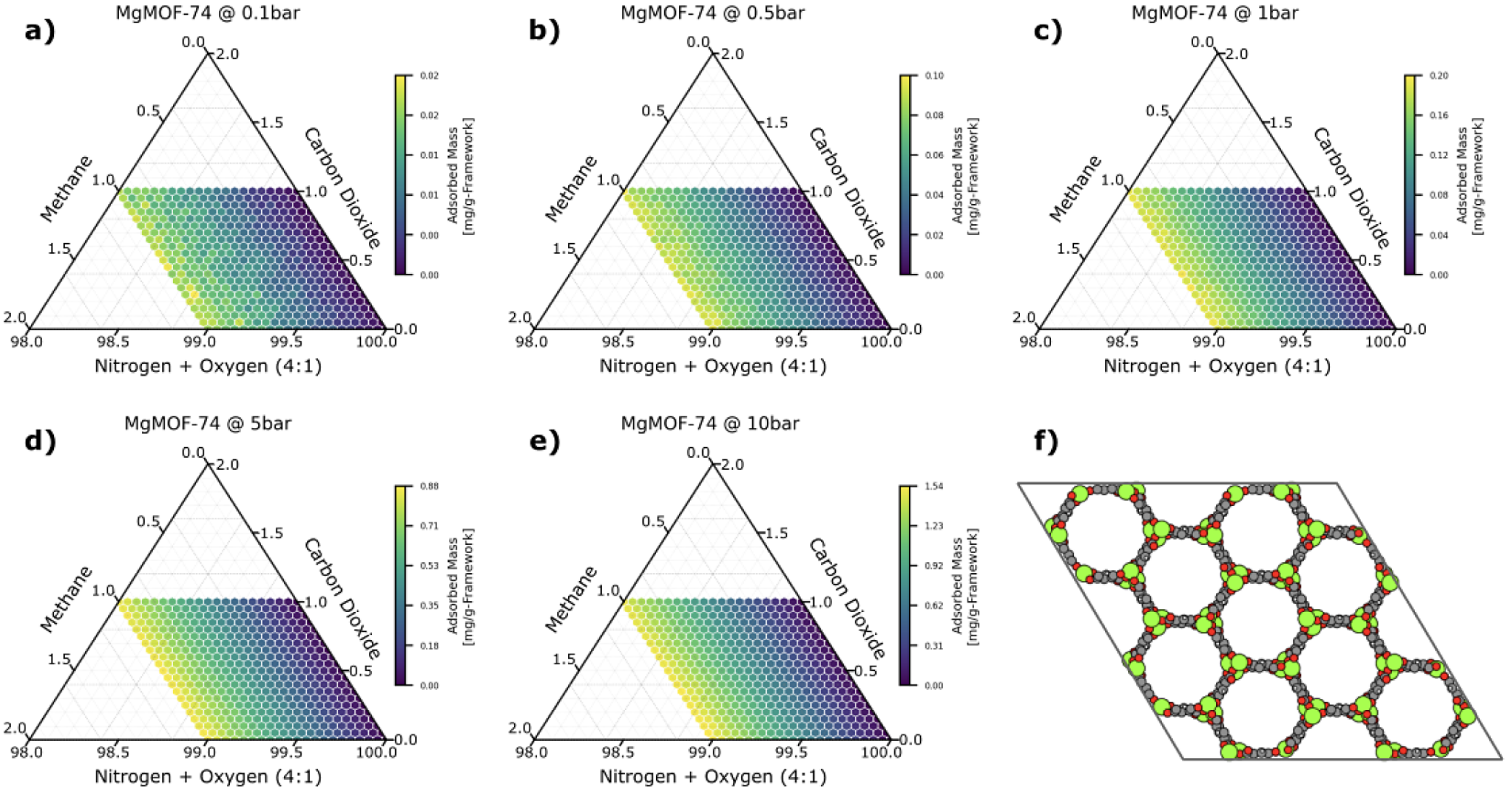
Ternary plots of the adsorbed mass of methane in MgMOF-74 as a function of composition and at the following pressures: a) 0.1 bar, b) 0.5 bar, c) 1 bar, d) 5 bar, and e) 10 bar. In panel f), we show a 2 × 2 × 2 unit cell of the MOF projected down the *c*-axis. Each axis shows the mole fraction of the corresponding gas (a 4:1 ratio for the nitrogen:oxygen axis), extending along the orientation of the tick marks. The color of each point in the ternary plot corresponds to the adsorbed mass of methane in units of mg/g-framework.

One of the MOFs which best demonstrates the ability of high-pressure sensing to improve small molecule detection by concentrating gases more strongly is the sensing material MgMOF-74, shown in Figure 1.^35^ As we increase the pressure of the system, the total adsorbed mass of hydrogen increases by 67-fold from 0.1 bar (0.0008 mg/g-framework) to 10.0 bar (0.0534 mg/g-framework). Similarly, the total adsorbed mass of methane, as shown in Figure 2, increases even more, with a 77-fold increase in mass from 0.1 bar (0.02 mg/g-framework) to 10.0 bar (1.54 mg/g-framework).

When the total adsorbed mass is too low, the mass detection limits of the device become significant, and measurement noise overwhelms the signal. Hence, the significant increase in the adsorbed mass from 0.1 to 10 bar is beneficial. Additionally, at higher pressures, simulation noise decreases, especially for weakly adsorbing gases, as evidenced by the smoothness of the high-pressure plots.

### Low Pressure Example (Benzene)

**Figure 3:**
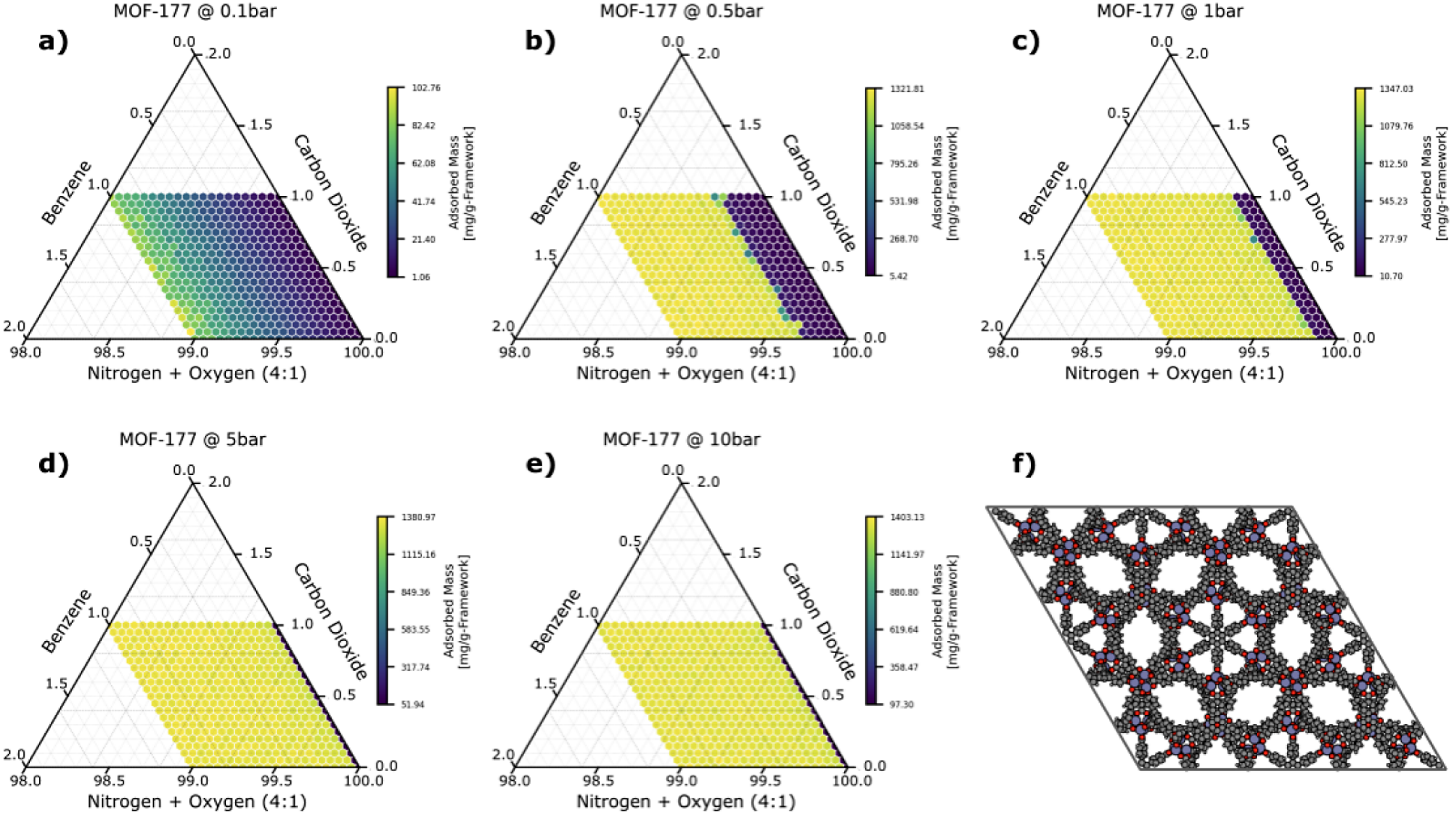
Ternary plots of the total adsorbed mass for benzene sensing in MOF-177 as a function of composition and at the following pressures: a) 0.1 bar, b) 0.5 bar, c) 1 bar, d) 5 bar, and e) 10 bar. In panel f), we show a 2 × 2 × 2 unit cell of the MOF projected down the *c*-axis. Each axis shows the mole fraction of the corresponding gas (a 4:1 ratio for the nitrogen:oxygen axis), extending along the orientation of the tick marks. The color of each point in the ternary plot corresponds to the total adsorbed mass in units of mg/g-framework.

Although nonpolar, benzene is generally a strongly adsorbing gas due to its large size. Even at low concentrations, it makes up a significant fraction of the total adsorbed mass in most MOFs and can rapidly saturate the sensor response (i.e., a change in the bulk concentration does not result in a change in the adsorbed mass). This not only makes difficult to detect benzene, but also to detect of nonbenzene gases, since the MOFs lose sensitivity towards those gases in the presence of benzene. To improve the detection of benzene, we hypothesized that it would be beneficial to decrease the pressure to the point that the sensor is no longer saturated, and changes in the benzene concentration would again result in a change of mass. We found that this effect does indeed occur and is demonstrated well using MOF-177 in Figure 3.^36^

Note that the maximum observed total adsorbed mass at 0.1 bar is significantly lower than that observed at 0.5 bar and above, and the rapid saturation of the MOF at pressures of 0.5 bar and above is consistent with the benzene isotherm for MOF-177, as shown in Figure S3. Even so, this decrease in the total adsorbed mass is coupled with the necessary desaturation of the sensor, enabling us to better distinguish ambient benzene concentrations over this range. Improving benzene sensing by shifting to low pressures highlights an important concept of the sensing elements of electronic noses; the best elements are those in which the *change* in the total adsorbed mass from one composition to another is greatest. It is easy to think that highly selective and highly adsorbing MOFs are best and, subsequently, that high mass loadings are universally desired. But as benzene demonstrates, this is not inherently true. At lower pressures, both the selectivity toward benzene and the total adsorbed mass decreases, but sensing is still improved because the change in mass as a function of change in composition is improved.

It should, however, be mentioned that for applications where benzene is present in extremely low concentrations (ppm and below), high pressures may not result in saturation of the sensing materials, and the array may actually benefit from high pressures due to a concentrating effect like that for hydrogen and methane. In fact, one of the MOFs we screened, NU-100, is more useful at high pressures when detecting benzene for this reason, and is discussed later.^37^ Nevertheless, all other MOFs screened perform best at low pressures, and Figure 3 demonstrates the potential benefits of low-pressure sensing.

### Multiple Pressure Example (Hydrogen Sulfide)

**Figure 4:**
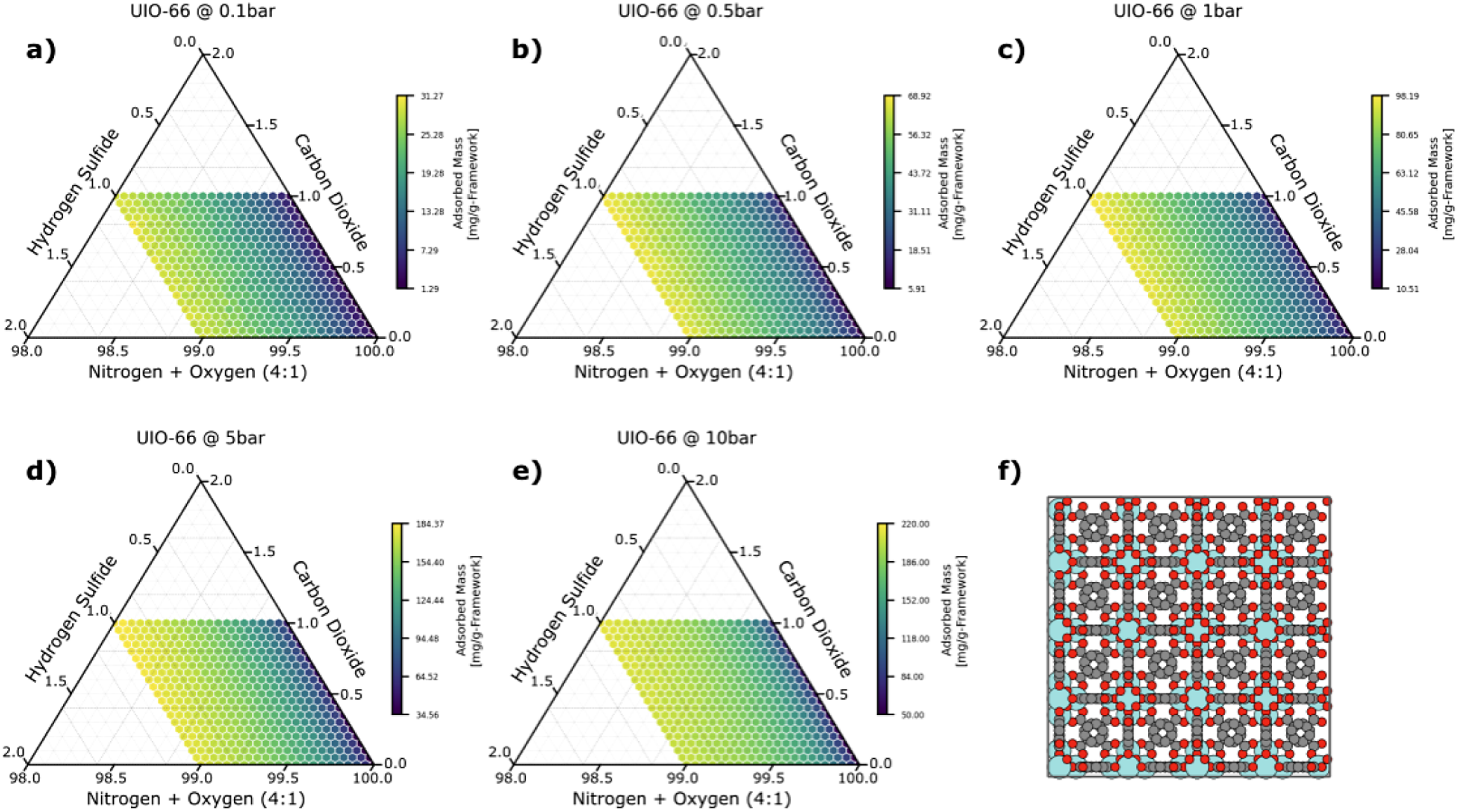
Ternary plots showing the adsorbed mass of hydrogen sulfide in UiO-66 as a function of composition and at the following pressures: a) 0.1 bar, b) 0.5 bar, c) 1 bar, d) 5 bar, and e) 10 bar. f) shows a 2 × 2 × 2 unit cell of the MOF projected down the *c*-axis. Each axis shows the mole fraction of the corresponding gas (a 4:1 ratio for the nitrogen:oxygen axis), extending along the orientation of the tick marks. The color of each point in the ternary plot corresponds to the adsorbed mass of hydrogen sulfide in units of mg/g-framework.

Hydrogen sulfide, like methane and hydrogen, is a small molecule, but also has a strong dipole moment that typically leads to stronger adsorption within MOF pores. Of the nine MOFs used in this study, several adsorb hydrogen sulfide appropriately for sensing at ambient pressure. Nevertheless, the adsorption behavior can still be beneficially modified by changing the system pressure. This is demonstrated well using UiO-66, as shown in Figure 4.^38^

At each of the simulated pressures, there is an appreciable change in the total adsorbed mass as a function of the composition, meaning that each pressure is useful for determining the composition of the gas mixture. At first glance, at high pressures and high concentrations of hydrogen sulfide (as seen in Figure 4d,e), the mass response as a function of composition appears to flatten out (the change in the adsorbed mass relative to the total adsorbed mass decreases). However, this apparent flattening of the response is sufficiently compensated by an overall increase in the total adsorbed mass, such that the absolute change in mass as a function of composition at high pressures is greater than at lower pressures, thus resulting in better sensing performance. Nevertheless, it is very likely that there are other MOFs that would benefit more from low-pressure sampling, when the increase in the total adsorbed mass at high pressures cannot compensate for this flattening behavior. Conversely, at low pressures and low concentrations of hydrogen sulfide, the change in mass in response to a change in composition is smaller, such that arrays using this MOF would benefit from high pressures.

For the nine MOFs screened in this work, hydrogen sulfide sensing, like hydrogen and methane sensing, is easier at high pressures. But unlike hydrogen or methane sensing, it is easy to envision an MOF for which the optimum sensing pressure is actually lower, especially if high concentrations of a gas are expected in the application, where saturation of the sensing material is more plausible.

## Discussion

**Figure 5:**
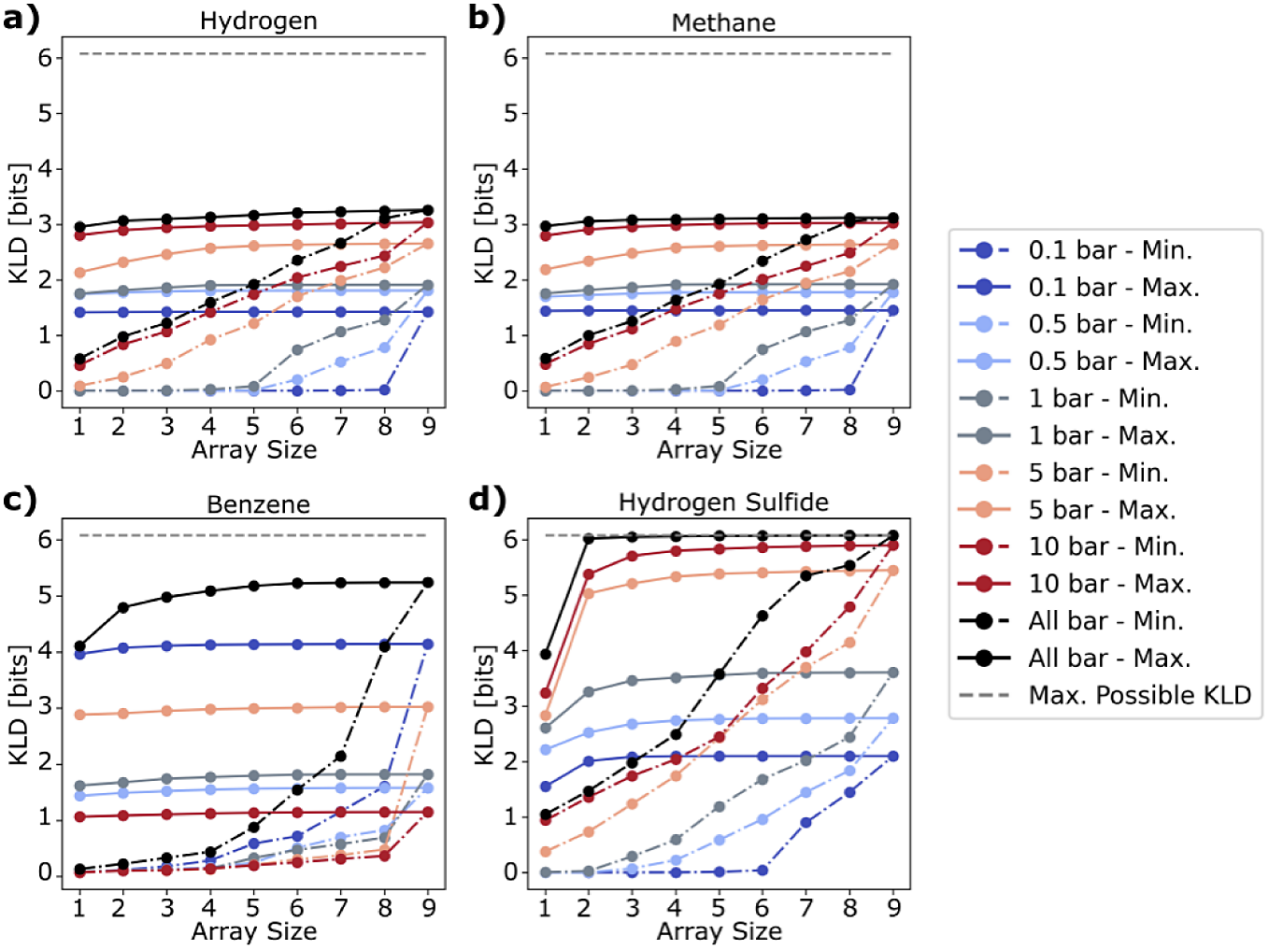
KLD vs pressure and array size for a) hydrogen arrays, b) methane arrays, c) benzene arrays, and d) hydrogen sulfide arrays. Solid lines are the best performing arrays (maximum KLD score), and dashed lines are the worst performing arrays (minimum KLD score). Note that with only nine MOFs, it is possible to test all arrays by brute force. The dashed gray line marks the maximum possible KLD score (6.089 bits) corresponding to a single composition with 100% probability.

Every possible array of each size and pressure was analyzed, and its performance was quantified with a KLD score. Figure 5 plots the best and worst array performance as a function of array size and operating pressure, including all-pressure arrays, in which the gas mixture was sampled at all five pressures rather than just one (black line).

In general, array performance always improves with array size; however, the change in the performance of the best arrays as a function of array size is sometimes minimal, suggesting that these arrays rely on only a few high-performing MOFs to make their predictions. However, we found that pressure often has a much more significant impact. For hydrogen, methane, and hydrogen sulfide containing gas mixtures, the array performance improves specifically with higher pressure operation. In fact, of these three gases, hydrogen sulfide is the only one that exhibits a significant increase in performance beyond size 1 arrays. The jump in performance from size 1 to size 2 arrays for hydrogen sulfide, especially at high pressures, suggests that increasing pressure results in an improvement in not just the individual adsorption behaviors but also the cross-sensitivity of the elements. Even then, beyond size 2 arrays, the improvement in performance is again minimal, consistent with the idea that in some circumstances, the best arrays need only a few elements.

Conversely, the worst arrays generally improve with both the pressure and array size. For example, the KLD scores of the worst arrays at 5 and 10 bar for hydrogen, methane, and hydrogen sulfide sensing all increase with the array size. This is because many of the MOFs exhibit useful adsorption behavior at these pressures. However, at low pressures, the increase in performance as a function of array size is limited as only a limited number of MOFs have any useful adsorption characteristics. For example, for hydrogen sensing at 0.1 bar, the KLD score for the worst arrays is nearly 0.0 until all nine MOFs are included in the array. This is because only one single MOF (MOF-801) has any useful adsorption characteristics for detecting hydrogen, as evidenced by the individual adsorption profiles of each MOF (Figure S4).

Together, these results highlight how varying pressure can change our approach toward both the search problem (i.e., screening MOFs) and array design problem (i.e., choosing the correct combination of MOFs) central to building an electronic nose. In terms of the search problem, for some gases, it will be easier to find materials with useful adsorption behaviors by examining fewer MOFs at more pressures, rather than more MOFs at a single pressure. Similarly, in terms of array design, using small arrays at an optimized pressure or set of pressures is more beneficial than using large arrays at a single unoptimized pressure. On this note, since methane, hydrogen, and hydrogen sulfide benefit specifically from high pressures, there is only a marginal improvement in the performance of the all-pressure arrays when compared to the single-pressure arrays operating at high pressures. However, this does not mean that there is never any benefit to operating at multiple pressures.

**Figure 6:**
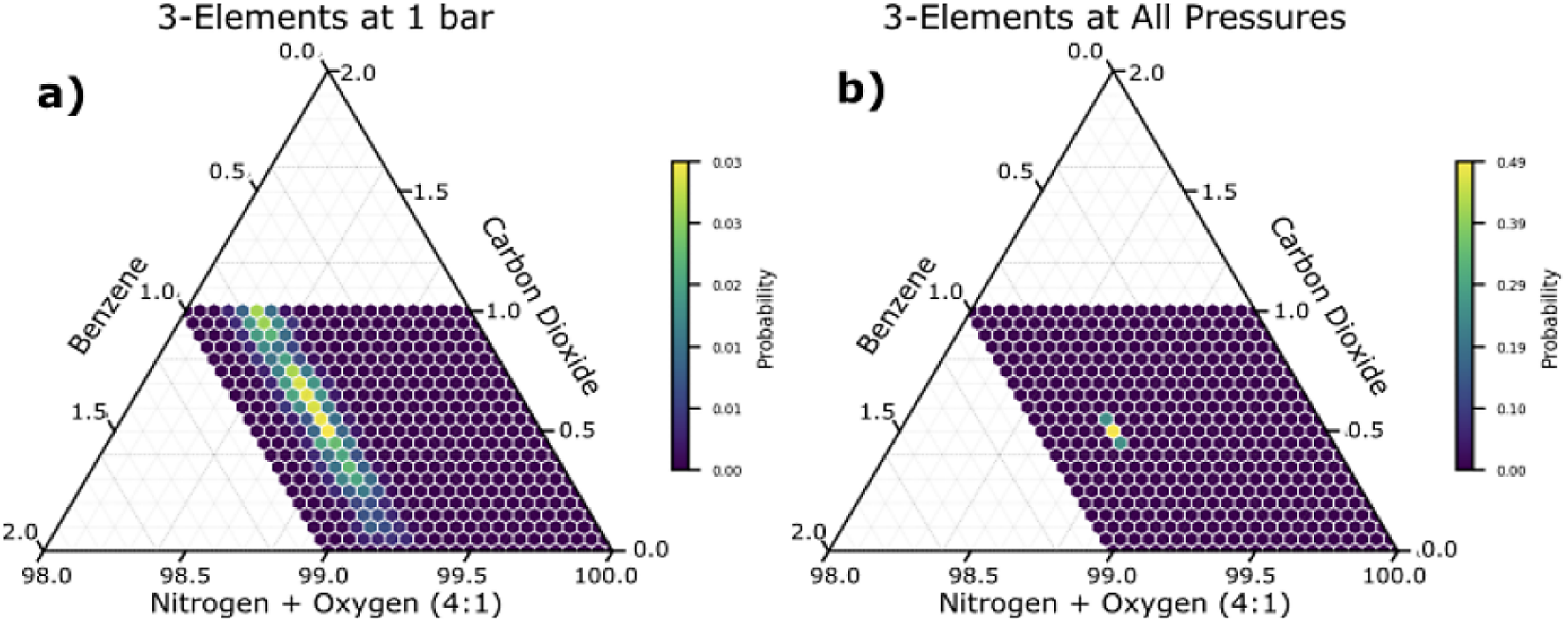
Probability vs. composition for a) the best three-element array at 1 bar (NU-100, MOF-177, and HKUST-1) and b) the best three-element array at all pressures (NU-100, IRMOF-1, and HKUST-1). These plots are generated by predicting the composition of an ‘unknown’ gas mixture given a set of detected masses (in this case, coming from the simulations of 0.5% carbon dioxide, 0.75% benzene, and balance of nitrogen and oxygen in a 4:1 mixture).

**Figure 7:**
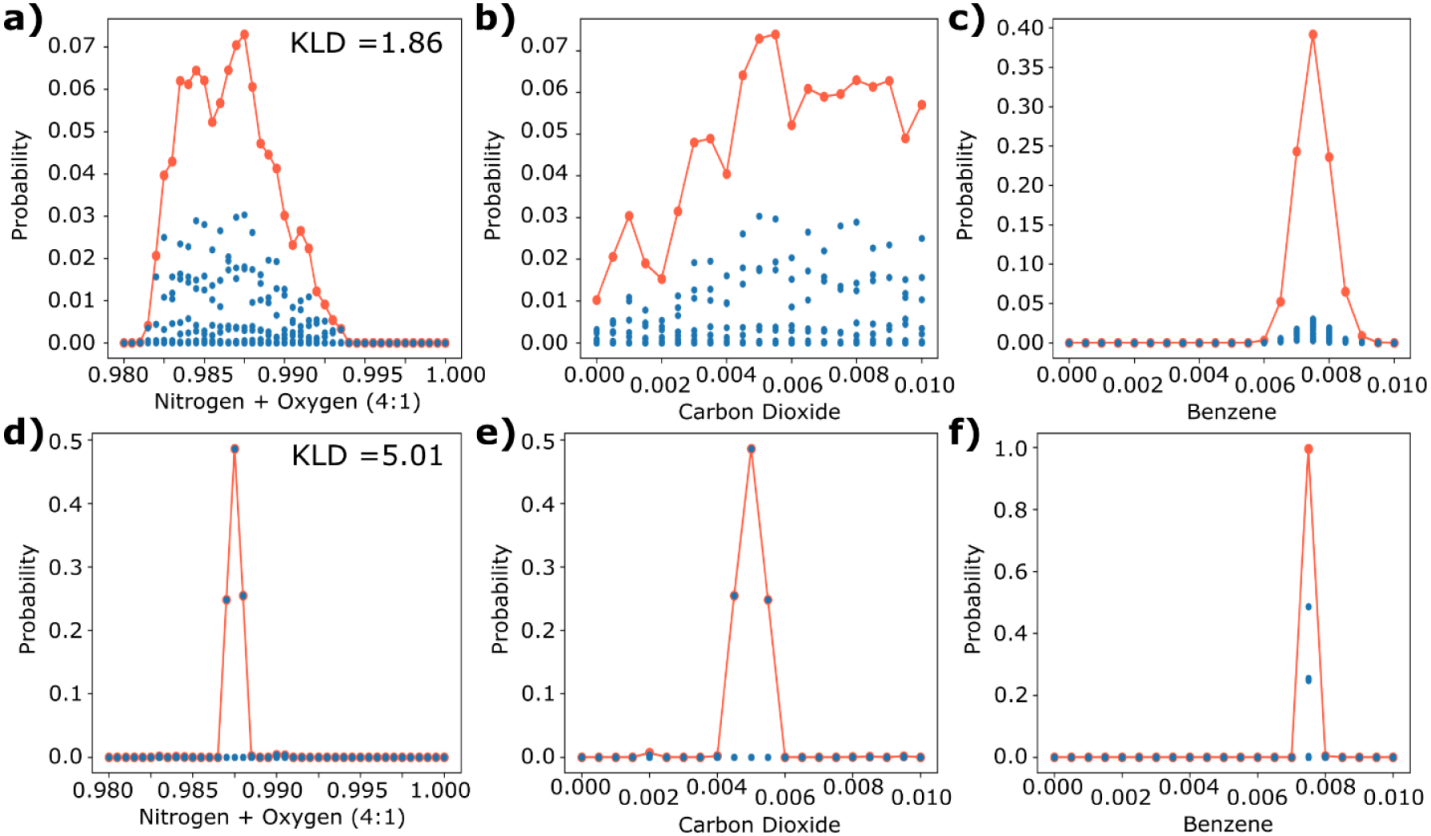
Probability vs component mole fraction for the best three-element array at 1 bar (NU-100, MOF-177, and HKUST-1) for a) nitrogen/oxygen, b) carbon dioxide, and c) benzene, and for the best three-element array at all pressures (NU-100, MOF-177, and HKUST-1) for d) nitrogen/oxygen, e) carbon dioxide, and f) benzene. These plots are generated by predicting the composition of an ‘unknown’ gas mixture given a set of detected masses (in this case, coming from the simulations of 0.5% carbon dioxide, 0.75% benzene, and balance of nitrogen and oxygen in a 4:1 mixture). Blue dots represent the probability of individual compositions, and the red line is the total probability for a component concentration. The all-pressure arrays include data at 0.1, 0.5, 1.0, 5.0, and 10.0 bar.

With benzene sensing, most of the MOFs at atmospheric and high pressures saturate at very low concentrations, making the detection of benzene beyond these concentrations practically impossible. By shifting to lower pressures, however, saturation occurs at higher benzene concentrations, thus enabling detection. Given this, one might expect benzene to benefit specifically from low-pressure sensing, just as hydrogen and methane benefited specifically from high pressures, but NU-100 exhibits unique behavior (Figure S2). It does not saturate at low benzene concentrations until operating at a pressure of 10 bar. At 5 bar, the change in mass as a function of benzene concentration is sharpest, making sensing at this pressure better than at either low or atmospheric pressures. In fact, the only single element that outperforms NU-100 at 5 bar is IRMOF-1 at 0.1 bar.^39^ As a result, there is a noticeable improvement in the performance of all-pressure arrays, with all of the best all-pressure arrays of size 2 or more containing NU-100 and IRMOF-1. When the composition of a gas mixture is predicted from the set of detected masses, the multiple pressure array noticeably outperforms the ambient pressure array. Figures 6a and 7a–c show the ternary probability plot and component probability plots of the best three-element array under ambient pressure (1 bar) sensing conditions, respectively. Figures 6b and 7d–f show the ternary probability plot and component probability plots of the best three-element array using multiple pressures, respectively.

It is clear from Figures 6 and 7 that just by sampling a few additional pressures, we can dramatically improve the ability to detect gases. Although the 1-bar array performs acceptably for the detection of benzene, there is still a wide margin of error, and the prediction for air and carbon dioxide is very poor. With multiplex sensing, we can narrow down the prediction to almost a single composition (i.e., all other compositions have a near-zero probability assigned to them).

While it is interesting that for benzene, some MOFs performed best at both low and high pressures, and consequently, there was a noticeable benefit for multiplex sensing, it is, in general, beneficial for sensing *mixtures* to consider multiple pressures. With most real gas mixtures being more complex than those used in this study, there will certainly be cases for which detecting certain species of gases benefits from lower pressures, while for others from high pressures, such as a system containing benzene and methane in air, which is relevant in natural gas processing.^40^

## Conclusion

For all gas mixtures, the studied MOF arrays showed improved performance at nonatmospheric pressures. Furthermore, the information gained from sampling at multiple pressures always resulted in improved performance when compared with sampling at just atmospheric pressure. The detection of hydrogen, methane, and hydrogen sulfide was specifically benefited from higher pressures (greater than 1 bar), whereas benzene detection was mostly benefited from lower pressures (less than 1 bar). Exceptionally, the MOF NU-100 performed best for benzene sensing at 5 bar, and thus, arrays for benzene sensing that contained NU-100 exhibited a notable improvement when sampling at multiple pressures. For most real gas mixtures, we speculate that sensing will benefit from leveraging both low and high pressures, as the gas mixtures will likely contain a combination of gases that are easier to detect at low pressures (e.g., benzene) and gases that are easier to detect at high pressures (e.g., hydrogen, methane, and hydrogen sulfide).

In general, low-pressure operation seems to benefit the detection of strongly adsorbing gases that easily saturate sensors, and high-pressure operation is better for detecting dilute or weakly adsorbing gases. By exploring and operating at multiple pressures, it is easier to both find useful MOF candidates and design cross-sensitive arrays. We have demonstrated an improvement in the sensing capabilities of the electronic noses while limiting the number of materials, making device fabrication cheaper and easier. Looking forward, we plan to combine our coefficient-based method for dilute gas adsorption with multiple pressure sampling introduced here.

## Supporting Information Available

The following files are available free of charge: Multiplex_Sensing_SI.pdf. Overview of computational methods, force field parameters, and additional adsorption plots (PDF)
Multiplex_Adsorption_Data.zip. csv files containing the raw simulated data for all MOFs, compositions, and pressures (ZIP)
