# Supplementary material for: Multipressure Sampling for Improving the Performance of MOF-based Electronic Noses
Source: ACS Sens. 2024 Jul 12;9(7):3531–9. doi: 10.1021/acssensors.4c00199 (PMC11287752; doi:10.1021/acssensors.4c00199)
Supplement: Supplementary file 1 — se4c00199_si_001.pdf [file se4c00199_si_001.pdf]

Multi-Pressure Sampling for Improving the Performance of MOF-based Electronic Noses

Brian A. Day[1], Nicolas I. Ahualli[1], and Christopher E. Wilmer[1,2,3*]

[1] Department of Chemical and Petroleum Engineering, University of Pittsburgh, 3700 O'Hara St, Pittsburgh, PA 15261, USA; brd84@pitt.edu
[2] Department of Electrical and Computer Engineering, University of Pittsburgh, 3700 O'Hara St, Pittsburgh, PA 15261, USA
[3] Clinical and Translational Science Institute, University of Pittsburgh, Forbes Tower, Meyran Ave Suite 7057, Pittsburgh, PA 15213
* Correspondence: wilmer@pitt.edu

Supporting Information - Table of Contents

1. RASPA Simulation Details
    - **Table S1**. Physical properties of MOF structures.
    - **Table S2.** Parameters of framework atoms.
    - **Table S3.** Parameters of gas molecule bodies.
    - **Table S4.** Critical parameters of gas molecules.
2. Composition Prediction & Array Analysis
    - **Figure S1.** Mapping from mass space to probability space for a ternary gas mixture of CO2, N2, and O2.
3. Additional Results

   3.1. Benzene Adsorption in NU-100
    - **Figure S2.** Ternary plots of the adsorbed mass of benzene NU-100 as a function of composition and at the following pressures: **a)** 0.1 bar, **b)** 0.5 bar, **c)** 1 bar, **d)** 5 bar, and **e)** 10 bar. **f)** shows a 2x2x2 unit cell of the MOF projected down the c-axis.

   3.2. Benzene Adsorption in NU-100
    - **Figure S3.** Isotherm for benzene adsorption in MOF-177 at 298K.

   3.3. Hydrogen Adsorption at 0.1 Bar
    - **Figure S4.** Ternary plots of the total adsorbed mass for hydrogen sensing at 0.1 bar as a function of composition. The MOFs shown are **a)** HKUST-1, **b)** IRMOF-1, **c)** MgMOF-74, **d)** MOF-801, **e)** MOF-177, **f)** NU-100, **g)** NU-125, **h)** UiO-66, and **i)** ZIF-8.
4. References

1. RASPA Simulation Details

Adsorption data was generated using RASPA, a grand canonical Monte Carlo simulation software designed by Dubbeldam et al.[1] We examined a set of 9 MOFs from the CoRe MOF database[2] and used a temperature of 298 K and pressures of 0.1, 0.5, 1, 5, and 10 bar. Simulations were conducted using 1000 initialization cycles and 5000 production cycles. A single cycle consists of *n* Monte Carlo steps, where *n* is equivalent to the number of molecules in the simulation. Note that this value fluctuates during a GCMC simulation. The simulations include the following moves: insertion, deletion, translation, regrowth (configuration is changed), and swapping.

To model electrostatic interactions, we assigned partial charges to the atoms of the MOF frameworks via the EQeq method.[3] Similarly, the molecule parameters of the gases also included partial charges, and the forcefield which we used, TrAPPE[4], has been shown to accurately simulate these effects.

Rigid MOF structures, as well as rigid molecule structures, were assumed, and Lennard-Jones (LJ) potentials with a cutoff of 12 Å were used along with Ewald charge interactions to determine the overall energy of the structure and adsorbed gases. The equations for LJ potential are given below, where ε is potential well-depth and σ is radius of interaction.

$$V_{ij} = 4\varepsilon_{ij}\left[\left(\frac{\sigma_{ij}}{r_{ij}}\right)^{12} - \left(\frac{\sigma_{ij}}{r_{ij}}\right)^{6}\right]$$

The equation for Ewald coulombic potential in a periodic system is given as:

$$U^{sys} = U^{real} + U^{rec}$$

$$U^{real} = \sum_{i<j} q_i q_j \frac{erfc(\alpha r_{ij})}{r_{ij}}$$

$$U^{rec} = \frac{2\pi}{V}\sum_{k}\frac{1}{k^2}e^{-\frac{k^2}{4\alpha^2}}\left(\left(\sum_{i=1}^{N} q_i \cos(k \cdot r_i)\right)^2 + \left(\sum_{i=1}^{N} q_i \sin(k \cdot r_i)\right)^2\right) - \sum_{i}\frac{\alpha}{\sqrt{\pi}}q_i^2$$

where q_i and q_j are the charges of particle i and j, respectively, $\boldsymbol{r_i}$ is the position of atom i, V is the volume of the cell, α is a damping factor, k is the wavelength, and erfc is the error function complement.

The information about each framework, including minimum number of unit cells, density, volumetric surface area, void fraction, and pore size (largest cavity diameter) are listed below in **Table S1**. Forcefield parameters (excluding partial charges, which are framework specific and can be found in the cif files) for each framework atom type are given in **Table S2**. Gas molecule parameters can be found in **Table S3**.

Table S1. Physical Properties of MOF Structures

MOF	Unit Cells [a, b, c]	Density [g/cm^3]	Surface Area [m^2/cm^3]	Void Fraction [---]	Pore Size [Å]
IRMOF-1	1, 1, 1	0.590375	2198.21	0.8108	15.08377
HKUST-1	1, 1, 1	0.879099	2114.54	0.7206	13.18983
NU-125	1, 1, 1	0.57834	2196.18	0.79	19.37323
UIO-66	2, 2, 2	1.22494	1762.62	0.6128	8.88
ZIF-8	2, 2, 2	0.924676	1442.14	0.6416	11.51766
MgMOF-74	1, 1, 4	0.91487	1549.21	0.6396	11.63962
MOF-177	1, 1, 1	0.426775	2035.73	0.8318	11.67849
NU-100	2, 2, 2	0.2843005	1620.675	0.8777	27.190265
MOF-801	1, 1. 1	1.74184	1303.21	0.5322	7.65165

Table S2. Parameters of Framework Atoms

Atom Type	/k_B [K]	[Å]
H	22.1417	2.886
C	52.8381	3.851
N	34.7222	3.66
O	30.1932	3.5
Mg	55.8574	2.69141
Cu	2.5161	3.495

Zn	62.3992	2.46155
Zr	34.7221	3.124

Table S3. Parameters of Gas Molecule Bodies

	Atom Type	X [Å]	Y [Å]	Z [Å]	/k_B [K]	[Å]	Charge [e]
**(0)**	O_CO_2	0.0	0.0	1.16	79.0	3.05	-0.35
**(1)**	C_CO_2	0.0	0.0	0.0	27.0	2.80	0.70
**(2)**	O_CO_2	0.0	0.0	-1.16	79.0	3.05	-0.35
**(0)**	N_N_2	0.0	0.0	0.55	36.0	3.31	-0.482
**(1)**	N_e*	0.0	0.0	0.0	---	---	0.964
**(2)**	N_N_2	0.0	0.0	-0.55	36.0	3.31	-0.482
**(0)**	O_O_2	0.0	0.0	0.605	49.000	3.02	-0.113
**(1)**	O_e*	0.0	0.0	0.0	---	---	0.226
**(2)**	O_O_2	0.0	0.0	-0.605	49.000	3.02	-0.113
**(0)**	H_H2	0.0	0.0	0.37	36.7	2.958	0.468
**(1)**	H_e*	0.0	0.0	0.0	---	---	-0.936
**(2)**	H_H2	0.0	0.0	-0.37	36.7	2.958	0.468
**(0)**	CH4_sp3	0.0	0.0	0.0	148.0	3.73	0.0
**(0)**	S_H2S	-0.3541	0.2743	-0.4189	122.0	3.60	0.0
**(1)**	H_H2S	0.9737	0.1714	-0.2619	50.0	2.5	0.21
**(2)**	H_H2S	-0.6197	-0.4457	0.6808	50.0	2.5	0.21
**(3)**	H2S_e*	-0.2465	0.1909	-0.2917	---	---	-0.42
**(0)**	C_benz	0.0	0.0	0.0	30.70	3.60	-0.095
**(1)**	C_benz	1.392	0.0	0.0	30.70	3.60	-0.095
**(2)**	C_benz	2.088	1.2055	0.0	30.70	3.60	-0.095
**(3)**	C_benz	1.392	2.411	-0.0012	30.70	3.60	-0.095
**(4)**	C_benz	0.0	2.411	-0.0017	30.70	3.60	-0.095
**(5)**	C_benz	-0.696	1.2055	-0.0007	30.70	3.60	-0.095
**(6)**	H_benz	-0.54	-0.9353	0.0004	25.45	2.36	0.095
**(7)**	H_benz	1.932	0.9353	0.0013	25.45	2.36	0.095
**(8)**	H_benz	3.1680	1.2055	0.0006	25.45	2.36	0.095
**(9)**	H_benz	1.932	3.3463	-0.0013	25.45	2.36	0.095
**(10)**	H_benz	-0.540	3.3463	-0.0026	25.45	2.36	0.095
**(11)**	H_benz	-1.776	1.2055	-0.0009	25.45	2.36	0.095

*X_e = mass-free region of charge

The Peng-Robinson equation of state, shown below, was used to calculate the fugacities necessary to run the GCMC simulation. The critical parameters for each molecule type are listed below in table S4.

$$p = \frac{RT}{V_m - b} - \frac{a\alpha}{V_m^2 + 2bV_m - b^2} \text{ where } a = \frac{0.457235R^2T_c^2}{p_c} \text{ \& } b = \frac{0.077796RT_c}{p_c}$$

$$\alpha = \left(1 + k(1 - T_r^{0.5})\right) \text{ where } k = 0.37464 + 1.54226\omega - 0.26992\omega^2 \text{ \& } T_r = T/T_c$$

Table S4. Critical Parameters of Gas Molecules

Molecule Type	T_C [K]	P_C [MPa]	ω	Bond Stretch
CO_2	304.1282	7.377300	0.22394	Rigid
N_2	126.192	3.395800	0.0372	Rigid
O_2	154.581	5.043000	0.0222	Rigid
H_2	33.19	1.315000	-0.214	Rigid
CH_4	190.564	4.599200	0.01142	---
H_2S	373.40	8.963000	0.09000	Rigid
C_6H_6	562.05	4.894000	0.2092	Rigid

N.B. The critical constants and the acentric factor for ammonia are from Perry's Chemical Engineering Handbook.[6]

2. Composition Prediction & Array Analysis

In order to assign probabilities to each composition, we compare the calculated set of masses at each composition (one per sensing element) to the set of sensor outputs. One element at a time, we take the sensor output associated with that element and create a truncated normal probability curve centered about the sensor output mass, with a fixed standard deviation value representative of measurement error. The reason for using a truncated probability distribution rather than a true normal distribution is to account for the fact that adsorption will always result in an increase in mass. Consequently, the lower bound is set at 0, and the upper bound is set at infinity.

The equations which govern the truncated normal distribution are as follows:

$$\varphi(\bar{\mu}, \bar{\sigma}, a, b; x) = 0 \; if \; x \leq a \,,$$

$$\varphi(\bar{\mu}, \bar{\sigma}, a, b; x) = \frac{\phi(\bar{\mu}, \bar{\sigma}; x)}{\Phi(\bar{\mu}, \bar{\sigma}^2; b) - \Phi(\bar{\mu}, \bar{\sigma}^2; a)} \; if \; a < b < x$$

$$\varphi(\bar{\mu}, \bar{\sigma}, a, b; x) = 0 \; if \; b \leq x$$

$$\phi(\bar{\mu}, \bar{\sigma}^2; x) = \frac{1}{\sigma\sqrt{2\pi}} e^{-\frac{(x-\mu)^2}{2\sigma^2}}$$

$$\Phi(\bar{\mu}, \bar{\sigma}^2; x) = \int_{-\infty}^{x} \frac{1}{\sigma\sqrt{2\pi}} e^{-\frac{(t-\mu)^2}{2\sigma^2}} dt$$

where $\phi(\bar{\mu}, \bar{\sigma}^2)$is the standard normal distribution over the interval $(-\infty, +\infty)$, and $\Phi(\bar{\mu}, \bar{\sigma}^2)$ is the cumulative distribution function over the interval $(-\infty, +\infty)$. The variables $\bar{\mu}$ and $\bar{\sigma}$ are the mean and variance of the parent normal distribution, and the variables a and b are the truncation interval.[12]

For each composition, we assign a probability based on where simulated mass sits on the truncated probability curve, as given by:

$$P_{sim,i} = \psi(\bar{\mu}, \bar{\sigma}, a, b; m_{sim,i})$$

where $\bar{\mu} = m_{exp}$, $\bar{\sigma} = 0.10$, $a = 0$, $b = \infty$. Since each mass is assigned a probability independently, the sum of all probabilities does not necessarily equal 1. However, since the intention of this process is to create a set of probabilities which will subsequently be used to calculate the array probabilities, we normalize the assigned probabilities for each sensing element so that now their sum equals 1. This guarantees that all sensing elements are given equal weight in the final prediction.

$$F = \sum_{i=1}^{N} P_{sim,i}$$

$$P_{sim,\,i}^{\,norm} = \frac{1}{F} \cdot P_{sim,i}$$

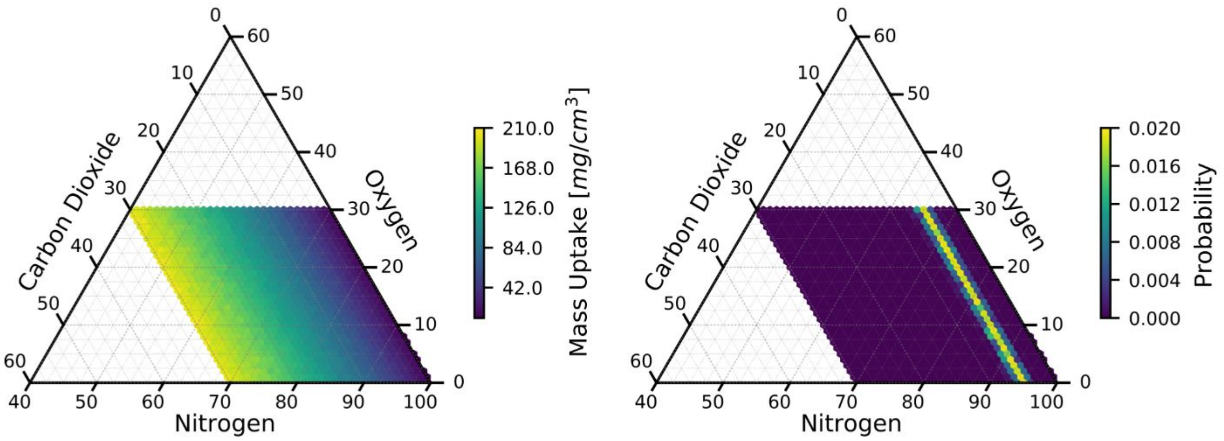

Figure S1. Mapping from mass space to probability space for a ternary gas mixture of CO2, N2, and O2.

Array performance was quantified with a metric known as the Kullbeck-Liebler divergence (KLD) score, which examines the difference between any two discrete sets of probabilities as follows:

$$KLD(P \parallel Q) = \sum_{i=1}^{N} P_i \cdot \log_2\left(\frac{P_i}{Q_i}\right)$$

where P_i and Q_i are the system and reference probability, respectively. The goal was to ask the question, "How much better are we predicting over random chance?", hence as a reference probability we used a simple uniform distribution, such that $Q_i = \frac{1}{N}$ for all i, with the above equation simplifying to:

$$KLD = \sum_{i=1}^{N} P_i \cdot \log_2(P_i \cdot N)$$

Note that we also dropped the $(P \parallel Q)$ notation, since our reference probability was never anything other than a uniform distribution.

3. Supplemental Results

3.1. Benzene Adsorption in NU-100

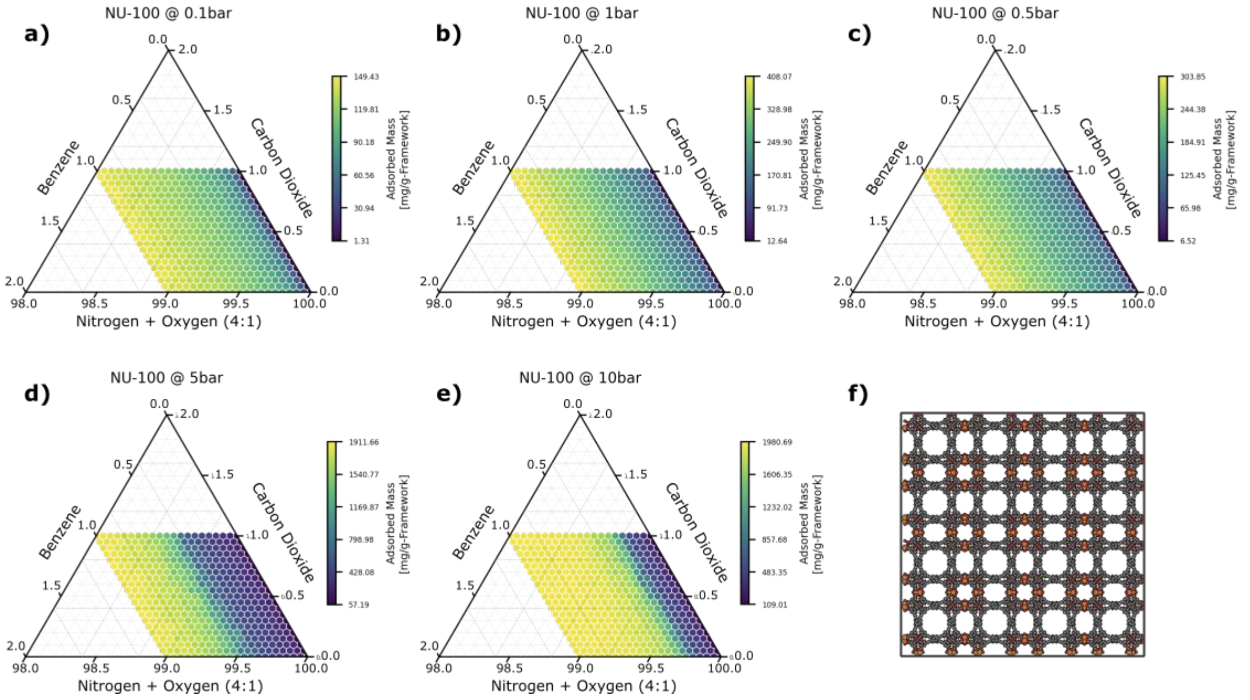

Figure S2. Ternary plots of the adsorbed mass of benzene NU-100 as a function of composition and at the following pressures: **a)** 0.1 bar, **b)** 0.5 bar, **c)** 1 bar, **d)** 5 bar, and **e)** 10 bar. **f)** shows a 2x2x2 unit cell of the MOF projected down the c-axis.

3.2. Benzene Isotherm for MOF-177

The sharp increase in the adsorbed concentration of benzene at approx. 10^3 Pa [0.01 bar] is consistent with the sharp increase in the adsorbion of benzene in the gas mixtures at a partial pressure for benzene of approx. 0.015 bar, most noticeable in Fig. 3b.

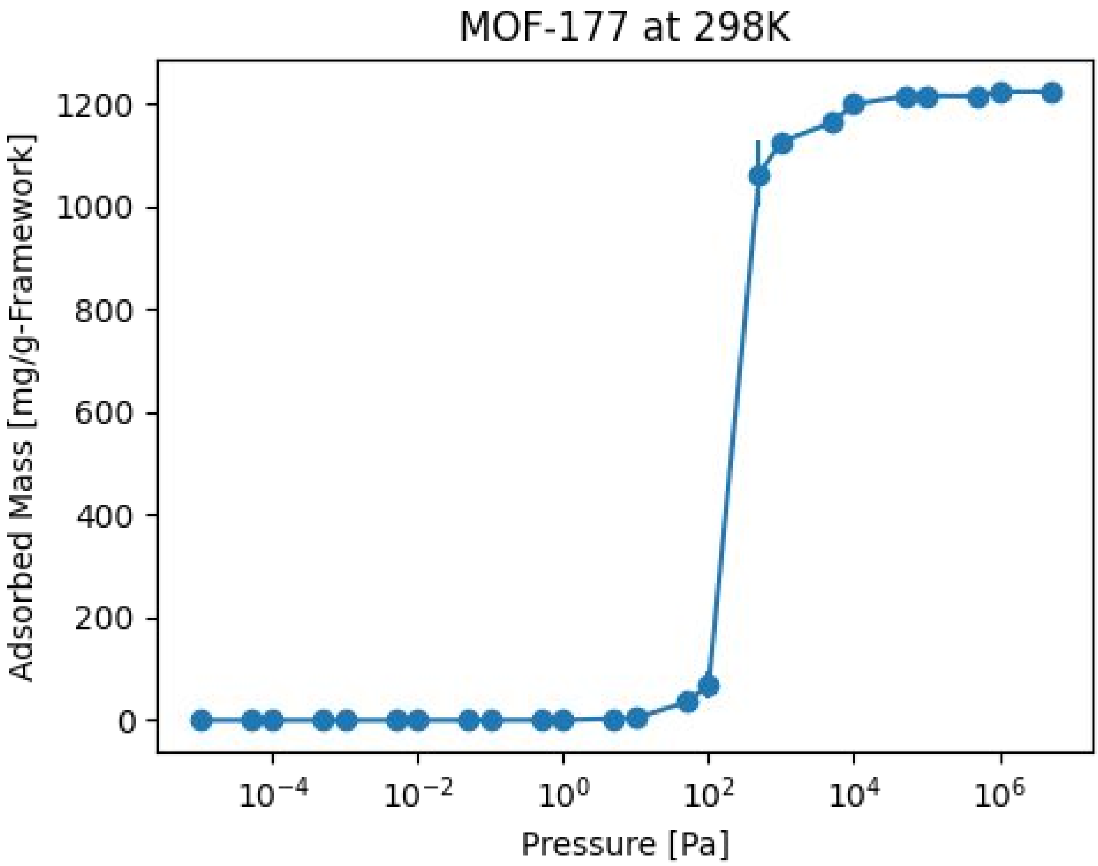

Figure S3. Isotherm for benzene adsorption in MOF-177 at 298K.

3.3. Hydrogen Adsorption at 0.1 Bar

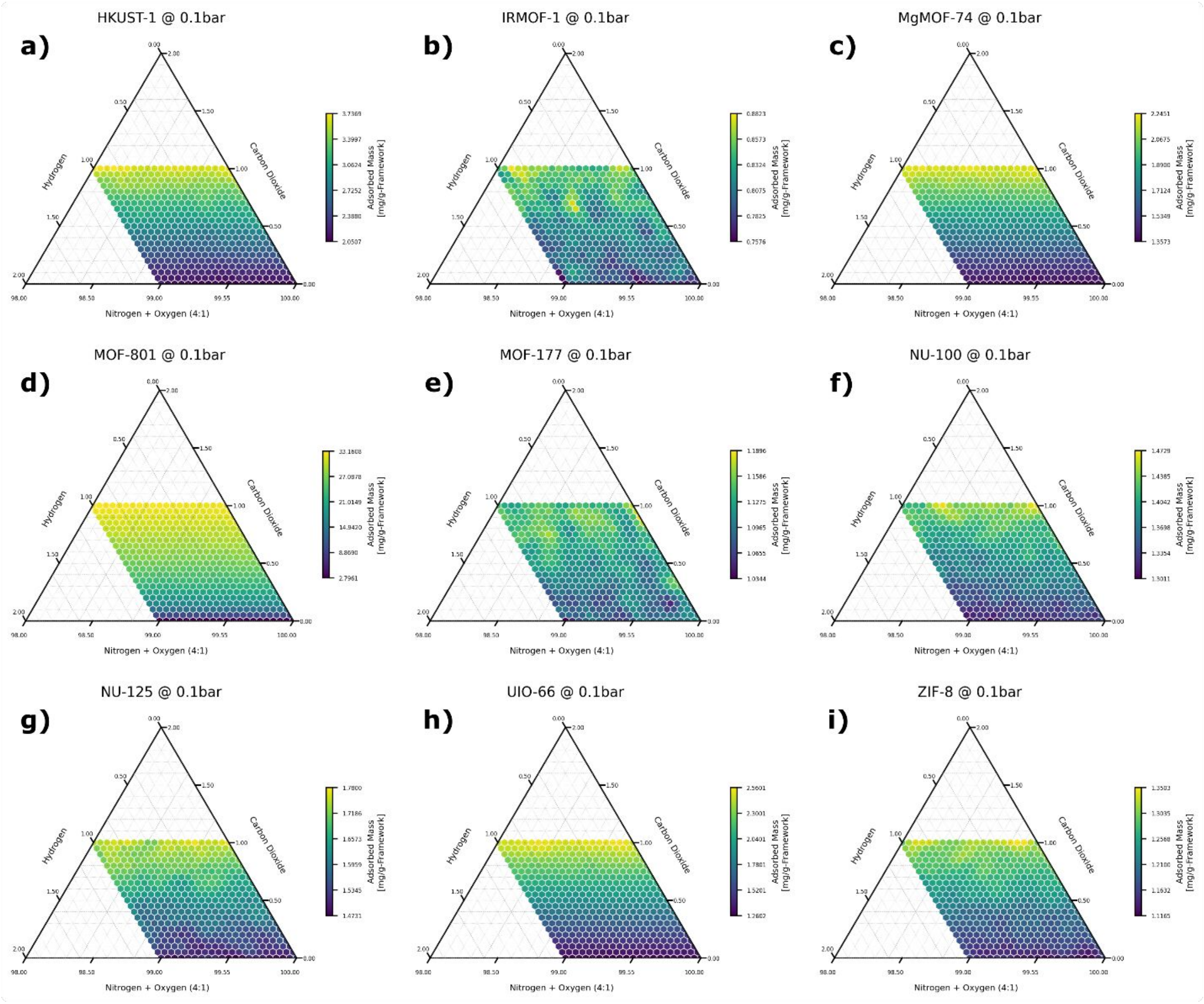

Figure S4. Ternary plots of the total adsorbed mass for hydrogen sensing at 0.1 bar as a function of composition. The MOFs shown are **a)** HKUST-1, **b)** IRMOF-1, **c)** MgMOF-74, **d)** MOF-801, **e)** MOF-177, **f)** NU-100, **g)** NU-125, **h)** UiO-66, and **i)** ZIF-8.

Because each of the figures is plotted on their own scale, at first glance it appears as though each MOF has enough variability to be a useful sensing material to some extent. However, upon closer examination, we can see that the difference in the total adsorbed mass

for many of the MOFs is quite small, especially compared to the simulated device error of 0.10 mg/g-framework. The total difference between the minimum and maximum adsorbed mass (in units of mg/g-framework) amongst all compositions for the MOFs are 1.6862 for HKUST-1, 0.1247 for IRMOF-1, 0.8878 for MgMOF-74, 30.3647 for MOF-801, 0.1552 for MOF-177, 0.1718 for NU-100, 0.3069 for NU-125, 1.2999 for UiO-66, and 0.2338 for ZIF-8. Only MOF-801 has a significant enough change in mass a function of composition to help predict the composition of the gas mixture, and even then, it is predominately useful for determining the composition of CO_2, and less useful for determining the composition of H_2.

4. References

(1) Dubbeldam, D.; Calero, S.; Ellis, D. E.; Snurr, R. Q. RASPA: Molecular Simulation Software for Adsorption and Diffusion in Flexible Nanoporous Materials. *Mol. Simul.* **2016**, *42* (2), 81–101. https://doi.org/10.1080/08927022.2015.1010082.

(2) Chung, Y. G.; Camp, J.; Haranczyk, M.; Sikora, B. J.; Bury, W.; Krungleviciute, V.; Yildirim, T.; Farha, O. K.; Sholl, D. S.; Snurr, R. Q. Computation-Ready, Experimental Metal–Organic Frameworks: A Tool To Enable High-Throughput Screening of Nanoporous Crystals. *Chem. Mater.* **2014**, *26* (21), 6185–6192. https://doi.org/10.1021/cm502594j.

(3) Wilmer, C. E.; Kim, K. C.; Snurr, R. Q. An Extended Charge Equilibration Method. *J. Phys. Chem. Lett.* **2012**, *3* (17), 2506–2511. https://doi.org/10.1021/jz3008485.

(4) Martin, M. G.; Siepmann, J. I. Transferable Potentials for Phase Equilibria. 1. United-Atom Description of n-Alkanes. *J. Phys. Chem. B* **1998**, *102* (14), 2569–2577. https://doi.org/10.1021/jp972543+.

(5) Eckl, B.; Vrabec, J.; Hasse, H. An Optimised Molecular Model for Ammonia. *Mol. Phys.* **2008**, *106* (8), 1039–1046. https://doi.org/10.1080/00268970802112137.

(6) Green, D. W.; Perry, R. H. *Perry's Chemical Engineers' Handbook, Eighth Edition*; McGraw Hill Professional, 2007.

(7) Farha, O. K.; Özgür Yazaydın, A.; Eryazici, I.; Malliakas, C. D.; Hauser, B. G.; Kanatzidis, M. G.; Nguyen, S. T.; Snurr, R. Q.; Hupp, J. T. De Novo Synthesis of a Metal–Organic Framework Material Featuring Ultrahigh Surface Area and Gas Storage Capacities. *Nat. Chem.* **2010**, *2* (11), 944–948. https://doi.org/10.1038/nchem.834.

(8) Gustafson, J. A.; Wilmer, C. E. Computational Design of Metal–Organic Framework Arrays for Gas Sensing: Influence of Array Size and Composition on Sensor Performance. *J. Phys. Chem. C* **2017**, *121* (11), 6033–6038. https://doi.org/10.1021/acs.jpcc.6b09740.

(9) Gustafson, J. A.; Wilmer, C. E. Optimizing Information Content in MOF Sensor Arrays for Analyzing Methane-Air Mixtures. *Sens. Actuators B Chem.* **2018**, *267*, 483–493. https://doi.org/10.1016/j.snb.2018.04.049.

(10) Gustafson, J. A.; Wilmer, C. E. Intelligent Selection of Metal–Organic Framework Arrays for Methane Sensing via Genetic Algorithms. *ACS Sens.* **2019**, *4* (6), 1586–1593. https://doi.org/10.1021/acssensors.9b00268.

(11) Day, B. A.; Wilmer, C. E. Genetic Algorithm Design of MOF-Based Gas Sensor Arrays for CO2-in-Air Sensing. *Sensors* **2020**, *20* (3), 924. https://doi.org/10.3390/s20030924.

(12) Burkardt, J. The Truncated Normal Distribution. Florida State University. https://people.sc.fsu.edu/~jburkardt/presentations/truncated_normal.pdf
